# Beyond Reading: Psychological and Mental Health Needs in Adolescents with Dyslexia

**DOI:** 10.3390/pediatric16040075

**Published:** 2024-10-15

**Authors:** Manuel-Ramón Morte-Soriano, Manuel Soriano-Ferrer

**Affiliations:** Department of Developmental and Educational Psychology, University of Valencia, 46010 Valencia, Spain

**Keywords:** dyslexia, attention-deficit/hyperactivity disorder, internalizing problems, externalizing problems, SDQ, teachers, parents, self-reports

## Abstract

Background. Overall, children and adolescents diagnosed with dyslexia or ADHD show an increased risk for psychological and mental health problems, and dyslexia and ADHD tend to coexist frequently. Thus, the main objective of this study was to examine psychological and mental health problems in dyslexia. Method. Participated 95 adolescents with dyslexia (DG), comorbid dyslexia + attention-deficit hyperactivity disorder, combined subtype (D + ADHD-CG), and a comparison group with typical development (TDCG). Self-reported measures of anxiety and depression, and parent and teacher versions of the Strengths and Difficulties Questionnaire (SDQ) were used. Results. Self-reports of internalizing problems showed that adolescents in the GD and D + ADHD-CG groups had more depression and stated anxiety problems with a very high percentage above the clinical cut-off point than the CG. Both the parent and teacher reports showed that the DG and D + ADHD-CG groups obtained higher mean values and a higher number of adolescents above the clinical cut-off of internalizing, externalizing, and total problems than the TDCG. The comorbid D + ADHD-CG group had the highest internalizing and externalizing problems. Conclusions. In conclusion, our findings indicate that the internalizing and externalizing problems experienced by adolescents with dyslexia and comorbid ADHD should be recognized early and treated promptly by education professionals.

## 1. Introduction

Achieving school-age reading proficiency is a public health issue as it is a good determinant of health and longevity and is associated with many indicators of academic, social, career, and even economic success [1,2,3]. However, the process of learning to read is fraught with difficulties and frustrations for some children. The latest edition of the Diagnostic and Statistical System of Mental Disorders [4] encompasses these difficulties known by the term dyslexia within “specific learning disorders”, which impede the ability to learn or use specific academic skills such as reading, writing, or arithmetic. Moreover, the difficulties are unexpected and cannot be explained by the lack of teaching, visual or auditory problems, or intellectual disability [4,5,6]. Two recent meta-analyses [7,8] indicated that this specific learning difficulty is very frequent, affecting around 7% of the world population, depending on the transparency of the orthographic system of the language and culture. Specifically in Spain, the prevalence has been estimated at 6% of secondary school students [9,10].

In addition to academic difficulties, students with dyslexia are at high risk for mental health problems, which commonly include measures of internalizing problems [11,12,13,14,15,16] as well as high levels of externalizing problems [12,13,17]. Traditionally, in child psychopathology [18], internalizing behavior refers to problems that occur primarily within oneself such as anxiety, depression, social isolation, and somatic complaints. On the other hand, externalized behavior includes conflicts with other people and is characterized by impulsivity, hyperactivity, aggressiveness, and rulebreaking.

Students with dyslexia may be more vulnerable to experiencing internalized disorders (e.g., anxiety and depression), possibly due to a low opinion of academic competence coupled with repeated experiences of academic failure [15,19,20]. For example, a meta-analysis of 42 studies revealed that about 70% of students with learning disabilities experienced greater anxiety symptoms than students without learning disabilities [21]. Another meta-analysis [22] conducted to understand depression among students with learning disabilities found higher scores on measures of depression than their peers without learning disabilities, which was true regardless of who reported depression-related symptoms (i.e., self-report, parents, or teachers). Similarly, some recent systematic review and meta-analysis studies [21,23,24] found that poor readers were at greater risk of experiencing anxiety and depression than normal learners with moderate to large effect sizes. In relation to depressive symptomatology, some studies [11,25,26] have found strong links between severe and persistent reading disabilities and an increased risk of depressive mood. However, other studies [27,28,29,30] have shown discrepant results, possibly due to inconsistencies and heterogeneity across studies [21,31]. The lack of significant differences between dyslexia and controls could be attributed to the age of the participants included in the studies. In line with this, a review of studies [32] found an increase in emotional symptomatology with increasing age in participants with dyslexia. Thus, adolescents with dyslexia presented a higher level of self-perceived anxiety, depression, and somatic symptoms while in childhood, no significant differences between dyslexics and controls appeared [33].

On the other hand, many studies have reported an increase in externalizing problems such as antisocial and aggressive behaviors among poor readers [12,16,20,34,35,36]. Externalizing behaviors and withdrawal behaviors can distract children from instruction and classroom activities, interfering with their ability to learn, leading to delayed reading acquisition and low achievement [37].

Different studies [38,39,40] have confirmed that students with dyslexia experienced significantly more internalizing, externalizing, and attention and behavioral problems according to the ratings of teachers, parents, and even the participants themselves. Many of these problems were even found to be above the clinical cut-off point [40].

In fact, learning disabilities such as dyslexia and attention-deficit hyperactivity disorder (ADHD) show high comorbidity [16,41]. In addition, students with ADHD also show high comorbidity with learning disabilities [42,43] and present greater internalizing and externalizing problems as reported by parents and teachers than their control peers [16,44,45,46].

In general, research has highlighted that children diagnosed with dyslexia or ADHD show an increased risk of internalizing and externalizing problems, and that dyslexia and ADHD tend to show high comorbidity [16]. For this reason, the comorbid group of dyslexia plus ADHD carries a higher risk of internalizing and externalizing problems than children with only dyslexia [31]. Therefore, the main aim of this study was to examine whether internalizing and externalizing problems varied in adolescents with dyslexia, with and without comorbid ADHD, and combined subtype using different sources of information such as self-reports as well as ratings of parents and teachers.

## 2. Materials and Methods

### 2.1. Participants

Ninety-five adolescents aged 12 to 15 years, together with their parents and teachers, participated in this study: 34 with dyslexia (DG), 28 with dyslexia + attention-deficit hyperactivity disorder (D + ADHD-CG), and 33 with typical development (TDCG). At the time of the study, all participants, from 13 secondary schools, lived in Spain and their mother tongue was Spanish. Table 1 shows the sociodemographic characteristics of all of the participants.

Dyslexia group (DG). Thirty-four adolescents with dyslexia, ranging in age from 12 to 15 years (mean age = 12 years and 10 months, SD = 0 year and 11 months; 79% male) participated. The DSM-5-TR [4] criteria specified to identify students with a specific learning disorder were used: (a) scores at or above 80 on an intelligence test [47], in order to exclude students with cognitive impairments; (b) absence of evidence or history of neurological damage, significant economic disadvantage, emotional disturbance, hearing or visual abnormalities, or any other significant disabling condition; and (c) a reading achievement score at or below the 25th percentile on the word and/or pseudoword reading skills subtests (accuracy and/or speed) of the Standardized Reading Skills Battery PROLEC-SE-R [48]. Two participants (5.9%) had been retained in a grade, 50% (n = 17) had received services in the school resource room prior to this study, and 79.4% (n = 27) had been reported by their teachers as needing help with their homework.

**Table 1:** Descriptive characteristics of adolescents and their families.

	DG (N = 34)	D + ADHD-CG (N = 28)	TDCG (N = 33)	Statistics		
	M (SD)	M (SD)	M (SD)	*F* _(2, 92)_	*p*	*η_P_* ^2^
Adolescent Age	12.88 (0.98)	13.19 (1.2)	12.71 (0.76)	1.75	0.17	0.03
Adolescent IQ	103.1 (8.9)	103.4 (12.4)	102.5 (15.5)	0.034	0.96	0.00
Age mother	42.2 (4.4)	44.9 (5.1)	44.5 (5.3)	2.91	0.06	0.06
Age father	46.3 (5.1)	47.4 (4.5)	47.6 (4.9)	0.76	0.29	0.02
**Adolescent Sex**	**n (%)**	**n (%)**	**n (%)**	***X*_(2)_^2^**	***p***	
Male	27 (79.4)	21 (75)	25 (75.8)	0.201	0.90	
Female	**7 (20.6)**	**7 (25)**	**8 (24.2)**			
**Marital Status**	**n (%)**	**n (%)**	**n (%)**	***X*_(2)_^2^**	***p***	
Married/living together	33 (97.1)	26 (92.9)	30 (90.9)	1.116	0.57	
Alone/widower/divorced	1 (2.9)	2 (7.1)	3 (9.1)			
**Mother’s occupation ^a^**	**n (%)**	**n (%)**	**n (%)**	***X*_(6)_^2^**	***p***	
Without paid job	8 (23.5)	0 (0)	8 (24.2)	28.72	0.001	
Skill level 1	17 (50)	8 (28.6)	14 (42.4)			
Skill level 2	7 (20.6)	20 (71.4)	6 (18.2)			
Skill level 3	2 (5.9)	0 (0)	5 (15.2)			
Skill level 4	0 (0)	0 (0)	0 (0)			
**Father’s occupation ^a^**	**n (%)**	**n (%)**	**n (%)**	***X*_(8)_^2^**	***p***	
Without paid job	0 (0)	5 (17.5)	0 (0)	24.78	0.002	
Skill level 1	3 (8.8)	0 (0)	5 (15.2)			
Skill level 2	24 (70.6)	23 (82.1)	19 (57.6)			
Skill level 3	6 (17.6)	0 (0)	7 (21.2)			
Skill level 4	1 (2.9)	0 (0)	2 (6.1)			

*Note.* **^a^** Based on the International Standard Classification of Occupations [49].

Dyslexia + Attention-Deficit Hyperactivity Disorder group (D + ADHD-CG). Twenty-eight adolescents participated, meeting both dyslexia and the combined subtype of ADHD, according to the DSM-5-TR criteria [4]. All adolescents had a previous diagnosis of ADHD combined subtype by mental health services, which was confirmed prior to their participation in this study. Thus, all of them presented the following criteria: six or more symptoms of inattention and six or more symptoms of hyperactivity-impulsivity according to parent and teacher assessments; persistence of symptoms for more than 6 months; and onset of symptoms before the age of 12 years. In addition, ADHD symptoms would interfere with the subject’s daily academic and/or social functioning. Exclusion criteria applied included having an IQ below 80, autism, psychosis, epilepsy, or any other neurological or genetic disease. The age range of the 28 adolescents in the D + ADHD-CG group ranged from 12 to 15 years (mean age = 13 years and 2 months, SD = 1 year and 2 months; 75% male). Less than 50% of the adolescents were receiving psychopharmacological (42.4%: n = 14) or psychological (36.4%; n = 12) treatment. Two participants (7.1%) had been retained in a grade, 67.9% (n = 19) had received services in the school resource room prior to this study, and 100% (n = 28) had been informed by the teacher that they needed help with their homework.

Typical Development Comparison Group (TDCG). Thirty-three adolescents in the typically developing control group attended regular classrooms and did not receive educational services outside their regular classroom. All participants in the CG group met the following criteria: (a) average or above average academic performance in all areas according to their teachers’ reports; (b) scores of 80 or above in intelligence; and (c) a reading achievement score equal to or above the 50th percentile on the word and pseudoword reading skills subtests of the Standardized Reading Skills Battery PROLEC-SE-R [48]. The age range of the 33 adolescents was between 12 and 15 years (mean age = 12 years and 8 months, SD = 0 year and 9 months. 75% male). None of the adolescents had been retained in a grade or attended the resource room. However, 6.1% (n = 2) had needed help with their homework. In some cases, information on the children’s academic and developmental history was obtained from the school’s psychoeducational support services, and in other cases, this information was obtained from the student’s family.

Demographic characteristics about the adolescents and families are presented in Table 1. There were no statistically significant differences between the three groups of adolescents in IQ and sex. In relation to the families, there were no statistically significant differences between the three groups in terms of parents’ age nor marital status. However, statistically significant differences were found in the mother’s occupation, ***X*_(8)_^2^** = 28.72, *p* < 0.001, and in the father’s occupation, ***X*_(8)_^2^** = 24.78, *p* < 0.002, so these variables were used as a covariate in the later analysis.

### 2.2. Measures

Intelligence. We used Factor g-R [47] to assess the general mental capacity without the interference of verbal stimuli. The reliability of the Spanish adaptation of this intelligence test is 0.93. In the current sample, the Cronbach’s alpha was 0.87.

Standardized Reading Skills Battery. Each adolescent’s word and pseudoword reading skills were measured with the Standardized Reading Skills Battery PROLEC-SE-R [48]. Word reading requires the correct identification of 96 words that vary greatly in frequency, length, and spelling structure, and pseudoword reading consists of pronouncing 48 pseudowords. In word and pseudoword reading, task completion is timed. These reading batteries had an adequate internal consistency with a Cronbach’s alpha > 0.95 for words and 0.77 for pseudoword reading in both the normalization sample and the current one.

Demographic Information Questionnaire. Parents supplied information about their sex, occupation [49], and marital status. They also responded to items about their child’s past and current schooling (e.g., number of grade retentions and whether they received help with their homework).

Anxiety. Adolescents completed the Spanish adaptation of the State-Trait Anxiety Inventory for Children STAIC; [50]. The STAIC distinguishes between a general proneness to anxious behavior rooted in personality (trait anxiety subscale) and anxiety as a fleeting emotional state (state anxiety subscale). Each subscale includes 20 Likert-type items, scoring on a 3-point scale ranging from 1 (almost never) to 3 (almost always). The Cronbach’s alpha is reported to be above 0.90 [50].

Depression. The Child Depression Inventory CDI; [51] is composed of 27 items that assess depressive symptomatology. The CDI assesses two scales: dysphoria (depressive mood, sadness, worry, etc.) and negative self-esteem (judgments of ineffectiveness, ugliness, meanness, etc.) and provides a total depression score. Each of the items has three response options that score 0 (no symptomatology), 1 (mild symptomatology), or 2 (severe symptomatology). Half of the items begin with the option that reflects the greatest severity of the symptom, and in the rest, the sequence of presentation is reversed. The score is obtained by adding each of the values attributed according to the child’s choice of response; therefore, the higher the score, the greater the intensity of depressive symptomatology presented, and a maximum score of 54 can be obtained. A score of 19 on the total scale represents the 90th percentile (a frequent cut-off point for screening depressed samples), and the scale usually has an alpha coefficient above 0.80 [51].

Emotional and Behavioral problems. The Spanish version of the Strengths and Difficulties Questionnaire (SDQ) for parents and teachers [52,53], which is designed to gather information about the emotional and behavioral difficulties experienced by children from 4 to 17 years old, was used. The SDQ queries positive and negative attributes displayed by the adolescents in the past 6 months across five subscales: emotional symptoms (e.g., often unhappy, down-hearted), conduct problems (e.g., often has temper tantrums or hot temper) and hyperactivity-inattention (e.g., restless, overactive, cannot stay still for long), peer relationship problems (e.g., tends to play alone), and prosocial behavior (e.g., considerate of other people’s feelings). Each subscale is measured by five items, rated by a 3-point scale ranging from 0 (strongly disagree) and 2 (strongly agree). To obtain the total scores, items are summed. Spanish normative data [54] set at the 90th percentile were used as the clinical cut-off range, except for the prosocial behavior subscale, where we used the 20th percentile. The test has been shown to have criterion validity and good test-retest reliability after four and six months (mean = 0.62). Furthermore, the internal consistency is satisfactory, with a Cronbach’s alpha ranging from 0.57 to 0.88 [52]. The Spanish adaptation [53] showed an internal consistency for total difficulties score of 0.84, ranging from 0.75 to 0.78 for the SDQ subscales. In the current sample, the Cronbach’s alpha was 0.96 for emotional problems, 0.92 for conduct and peer relationship problems, and 0.82 for hyperactivity-inattention and prosocial behavior.

### 2.3. Data Analysis

Data were analyzed using SPSS version 24. First, descriptive statistics were used in order to describe the characteristics of the participants (see Table 1). Therefore, in the case of continuous variables, the mean and SD are provided, while frequencies and percentages are provided for categorical variables. In order to compare psychological and mental health problems between groups, after verifying that the data fulfilled the criterion of statistical normality, applying the Kolmogorov–Smirnov test, a multivariate analysis of covariance (MANCOVA) was performed with the parents’ occupations as covariates (mothers and fathers) due to the statistically significant differences between groups (see participants section) and the group of origin (DG, D + ADHD-CG, TDCG) as a grouping factor. Next, a between-groups analysis of covariance (ANCOVA) was conducted for each measure with the parents’ occupations as covariates (mothers and fathers). To control for multiple comparisons, a Bonferroni correction (0.05/17 = 0.002) was applied in order to determine the significance levels. We report the effect sizes using the partial eta squared statistic (η_p_^2^) with values between 0.01 and 0.10 considered small effect sizes, values between 0.10 and 0.49 considered medium, and values above 0.50 considered large effect sizes. Subsequently, the Bonferroni post hoc test was applied to check the differences between pairs of groups. Additionally, we computed the percentage of adolescents in each group who obtained scores in the clinically elevated range. To do this, dichotomous variables were created to analyze clinically significant impairments of the participants. Each measure was coded as 0 (below 90th percentile) and 1 (above 90th percentile), except for the SDQ prosocial behavior subscale, where we used the 20th percentile.

## 3. Results

The MANCOVA performed with the parents’ occupations as covariates showed significant main effects by group (Wilks’ Lambda (Λ) = 1197, *F*_(32, 152)_ = 7.081, *p* < 0.001, *η_p_*^2^ = 0.60), with a large effect size.

In relation to mental health problems, results of the ANCOVAs showed significant differences between groups in the majority of variables (see Table 2). Thus, the results showed significant differences between groups in dysphoria, *F*_(2, 90)_ = 37.604, *p* < 0.001, *η_p_*^2^ = 0.45, and in negative self-esteem, *F*_(2, 90)_ = 21.953, *p* < 0.001, *η_p_*^2^ = 0.32, total depression, *F*_(2, 90)_ = 35.687, *p* < 0.001, *η_p_*^2^ = 0.44, and state anxiety, *F*_(2, 90)_ = 30.101, *p* < 0.001, *η_p_*^2^ = 0.40, with a medium effect size. The Bonferroni post hoc showed that in all cases, the DG and D + ADHD-CG groups had more internalizing problems than the other group (TDCG). In addition, there were no significant differences between the DG group and the D + ADHD-CG group. As shown in Table 2, 58.8% in the DG and 60.7% in the D + ADHD-CG groups scored above the clinical cut-off point (above the 90th percentile) in total depression. On the other hand, between 88.2% in DG and 100% of adolescents in the D + ADHD-CG group were identified as clinically impaired in state anxiety (above the 90th percentile). In relation to trait anxiety, the results of the ANCOVAs did not show significant differences between groups. Nevertheless, around 12% of adolescents in the DG and 14% in the D + ADHD-CG groups were identified as clinically impaired in trait anxiety (above 90th percentile), whereas 0% were identified in the TDCG (see Table 3).

In relation to the parents’ ratings of internalizing and externalizing problems, results of the ANCOVAs showed significant differences between groups in the majority of variables (see Table 4). Therefore, the results showed significant differences between groups in emotional symptoms, *F*_(2, 90)_ = 20.746, *p* < 0.001, *η_p_*^2^ = 0.31 and in conduct problems, *F*_(*2, 90)*_ = 8.227, *p* < 0.001, *η_p_*^2^ = 0.15, with a medium effect size, and in inattention-hyperactivity, *F*_(2, 90)_ = 62.100, *p* < 0.001, *η_p_*^2^ = 0.58 and total difficulties, *F*_(2, 90)_ = 45.650, *p* < 0.001, *η_p_*^2^ = 0.50 with a large effect size. The Bonferroni post hoc showed that in all cases, the D + ADHD-CG group had more emotional and behavioral problems than the other two groups (DG and CG). Additionally, the DG group also showed significantly more emotional and behavioral problems than the CG, with the exception of conduct problems, where there were no differences between the DG and TDCG groups. As indicated in Table 5, the parents reported as clinically impaired in emotional symptoms was 50% of D + ADHD-CG, 24% of DG, and 3% of TDCG; in terms of conduct problems and the total scale, 18% of D + ADHD-CG, 12% of DG, and 0% in TDCG were identified as clinically impaired. Finally, in the inattention-hyperactivity subscale, 96.4% of D + ADHD-CG and 56% of DG were identified by their parents as clinically impaired.

In relation to peer problems and prosocial behaviors, the results of ANCOVAs did not show significant differences between groups. Nevertheless, in peer problems, around 43% of adolescents in the D + ADHD-CG group were identified as clinically elevated by their parents, whereas parents identified around 10% in peer problems in the DG and TDCG (see Table 5). Additionally, the parents reported more adolescents with less prosocial behaviors in the D + ADHD-CG (29%) group than in the others two groups (0% in DG and 6% in TDCG).

With regard to the teachers’ ratings of emotional and behavioral problems, the results of the ANCOVAs showed significant differences between groups (see Table 6) in emotional symptoms, *F*_(2, 90)_ = 27.176, *p* < 0.001, *η_p_*^2^ = 0.38 and in conduct problems, *F*_(2, 90)_ = 26.347, *p* < 0.001, *η_p_*^2^ = 0.37 with a medium effect size, and in inattention-hyperactivity, *F*_(2, 90)_ = 137.95, *p* < 0.001, *η_p_*^2^ = 0.75 and total difficulties, *F*_(2, 90)_ = 13.642, *p* < 0.001, *η_p_*^2^ = 0.65 with a large effect size. The Bonferroni post hoc showed that in all cases, the D + ADHD-CG group had more emotional and behavioral problems than the other two groups (DG and TDCG). Additionally, the DG group also showed significantly more emotional and behavioral problems than the TDCG, with the exception of conduct problems, where there were no differences between the DG and TDCG groups.

As indicated in Table 5, teachers reported 75% of D + ADHD-CG, 40% of DG, and 0% of TDCG as clinically impaired in emotional symptoms. In the conduct problems subscale, 50% of the D + ADHD-CG were identified in the clinical range, while only 3% of DG and 0% of TDCG were classified as clinically impaired. In the total scale, again more than 60% of the D + ADHD-C group was reported by teachers to be clinically impaired compared to 5% of DG and 0% of TDCG. Finally, in the inattention-hyperactivity subscale, 100% of the D + ADHD-CG group were identified by teachers as clinically impaired, whereas only 20% of DG were in the clinical range.

In relation to peer problems and prosocial behaviors, the results of the ANCOVAs did not show any differences between groups. Nevertheless, in peer problems, around 25% of adolescents in the D + ADHD-CG group were identified as clinically elevated by their teachers, whereas the parents identified around 15% in peer problems in the DG and 10% in the TDCG groups (see Table 5). Additionally, the teachers reported slightly more adolescents with less prosocial behaviors in the D + ADHD-CG group (10%) than in the other groups (6% in DG and 0% in TDCG).

## 4. Discussion

To our knowledge, this study is the first to examine internalizing and externalizing problems in Spanish adolescents with dyslexia, with and without ADHD, using different sources of information such as self-reports and the ratings from the parents and teachers.

In general, both the self-reports from the adolescents themselves and the parents and teachers reported that Spanish adolescents in the two groups with reading difficulties (DG and D + ADHD-CG) obtained higher mean values of internalizing (e.g., state anxiety, depression, emotional problems), externalizing (e.g., conduct problems, inattention-hyperactivity), and total problems than the comparison group, with a higher number of adolescents above the clinical cut-off. In addition, we confirmed that the sample size was adequate and that the power of the significant results was adequate (range 0.80 to 0.83) by using G*Power 3 [55]. Our results are consistent with those in previous studies showing dyslexia as a risk condition for internalizing and externalizing problems [11,13,14,15,16,18,19,21,22,24,25,34,35,36,37,38,39,40], especially during adolescence [31,32].

One aspect that deserves to be highlighted from our findings is that adolescents in the D + ADHD-CG group presented the greatest internalizing and externalizing problems according to the ratings of the parents and teachers, since, as concluded in the review of previous studies, comorbidity with ADHD is one of the main risk factors [31]. However, our findings with the adolescent self-reporting measures failed to show differences between the two groups with reading difficulties, with and without associated ADHD (GD and D + ADHD-CG). This lack of differences between the two groups is possibly due to strong evidence that a substantial proportion of students with attention-deficit hyperactivity disorder (ADHD) tend to make more positive assessments of their own abilities relative to other external assessments. That is, children and adolescents with ADHD appear to exhibit a self-enhancement bias, being unaware of their thinking patterns, feelings, and behaviors, so their self-reports are often at variance with the assessment of their parents and teachers of them, even with objective evaluations. This positive self-assessment may persist into adulthood [56,57,58]. Although these discrepancies between self-reports and parent and teacher ratings may be considered as measurement errors or informant bias [59], they may also reflect the true differences related to the environment (e.g., home and school) and/or differences in the informant’s knowledge and experiences [60,61,62,63,64].

Our results should be interpreted in light of some of the limitations of the present study. A first limitation concerns the age range of the participants. Our sample was limited to early adolescence (13–15 years), so further research should investigate in depth the development of mental health problems in older adolescents. Longitudinal studies would allow for the detection of early signs of emotional and behavioral problems as well as the possible risk and protective factors for these problems and their progression through the life cycle. A second limitation was the absence of an ADHD group without a reading disability. Comparing this group with an ADHD group without dyslexia would allow us to better understand the impact of ADHD on internalizing and externalizing symptoms.

There are several implications for the practice of our findings. Our results confirm that the presence of dyslexia increases the risk of experiencing mental health problems (anxiety and depression) as well as the presence of internalizing and externalizing problems. Some researchers [12,13] have highlighted that most interventions tend to focus on academic domains, not paying attention to internalizing and externalizing problems. Given that academic progress, emotional well-being, and mental health are intimately related [12,19,65], the presence of mental health problems should be promptly recognized and treated in combination with academic problems, especially in school contexts [66,67] due to several reasons: (a) adolescents spend a large number of hours per day in schools; (b) mental health problems can be normalized to a greater extent due to the number of students; and (c) they allow for easy access to teachers and families who can participate in the implementation of interventions. Recent reviews [65,66,67] have confirmed the effectiveness that different psychoeducational interventions, many of which employ cognitive-behavioral techniques, have in reducing the risk and/or symptomatology of anxiety and depression in adolescents. For example, the solution focused behavior therapy program [68,69] focuses on enhancing the behavioral and social-emotional functioning of students with reading difficulties and/or ADHD as well as their academic achievement. In the same vein, other studies [70,71] have confirmed the effectiveness of combined programs on reading and anxiety, both in terms of improving reading difficulties and comprehension as well as reducing anxiety symptoms.

## 5. Conclusions

In summary, interventions aimed at students with learning difficulties such as dyslexia should address both academic problems and their mental health and/or socio-emotional and behavioral problems, in order to provide them with an educational response more appropriate to their needs. Moreover, if these combined interventions are developed in the early grades, they could further reduce both the academic difficulties and internalizing and externalizing problems of adolescents.

## Figures and Tables

**Table 2:** Comparison of mental health (depression and anxiety) problems.

	DG (N = 34)		D + ADHD-CG (N = 28)		TDCG (N = 33)		F_(2, 90)_	*p*	*η_p_* ^2^
	M	SD	M	SD	M	SD			
**CDI—Dysphoria**	6.62	2.36	7.39	2.37	2.45	2.30	37.604	0.001	0.45
**CDI—Negative Self-Esteem**	10.62	2.72	11.04	2.70	6.82	3	21.953	0.001	0.32
**CDI—Total Depression Score**	17.24	4.20	18.46	4.70	9.27	4.95	35.687	0.001	0.44
**STAIC—Trait Anxiety**	36.21	7.45	36.07	7.69	32.21	5.04	4.637	0.012	0.09
**STAIC—State Anxiety**	47.85	10.02	53.21	5.23	36	8.04	30.101	0.001	0.40

**Table 3:** N and percentage of adolescents above a clinically elevated score.

	DG (N = 34)	D + ADHD-CG (N = 28)	TDCG (N = 33)
	n (%)	n (%)	n (%)
**CDI—Total Depression Score**	20 (58.8)	17 (60.7)	2 (6.1)
**STAIC—Trait Anxiety**	4 (11.8)	4 (14.3)	0 (0)
**STAIC—State Anxiety**	30 (88.2)	28 (100)	3 (9.1)

**Table 4:** Comparison of the parents’ ratings on emotional and behavioral problems.

	DG (N = 34)		D + ADHD-CG (N = 28)		TDCG (N = 33)		*F*_(2, 90)_	*p*	*η_p_* ^2^
SDQ	M	SD	M	SD	M	SD			
**Emotional Symptoms**	2.68	2.5	4.4	2.2	0.88	1.08	20.746	0.001	0.31
**Conduct Problems**	1.32	1.47	1.54	1.5	0.52	0.56	8.227	0.001	0.15
**Inattention-Hyperact.**	4.62	2.4	7	1.33	1.58	1.6	62.100	0.001	0.58
**Peer Problems**	1.82	2.4	2.29	2.1	0.94	1.4	2.673	0.074	0.05
**Prosocial Behaviors**	8.44	3.1	8.4	1.3	8.70	1.4	0.192	0.825	0.00
**Total Difficulties**	9.85	5.51	15.11	4.4	3.91	3.5	45.650	0.001	0.50

**Table 5:** N and percentage of the ratings of the parents and teachers above a clinically elevated score in each group.

	DG (N = 34)		D + ADHD -CG (N = 28)		TDCG (N = 33)	
	Parent	Teacher	Parent	Teacher	Parent	Teacher
	n (%)	n (%)	n (%)	n (%)	n (%)	n (%)
**Emotional Symptoms**	8 (23.5)	14 (41.2)	14 (50)	21 (75)	1 (3)	0 (0)
**Conduct Problems**	4 (11.8)	1 (2.9)	5 (17.9)	14 (50)	0 (0)	0 (0)
**Inattention-Hyperact.**	19 (55.9)	7 (20.6)	27 (96.4)	28 (100)	1 (3)	0 (0)
**Peer Problems**	4 (11.8)	5 (14.7)	12 (42.9)	7 (25)	3 (9.1)	3 (9.1)
**Prosocial Behaviors**	0 (0)	2 (5.8)	8 (28.6)	3 (10.3)	2 (6.1)	0 (0)
**Total Difficulties**	4 (11.8)	2 (5.2)	5 (17.9)	18 (64.3)	0 (0)	0 (0)

**Table 6:** Comparison of the teachers’ ratings on emotional and behavioral problems.

	DG (N = 34)		D + ADHD-CG (N = 28)		TDCG (N = 33)		*F*_(2, 90)_	*p*	*η_p_* ^2^
	M	SD	M	SD	M	SD			
**Emotional Symptoms**	2.1	2.1	4.04	1.9	0.76	1	27.176	0.001	0.38
**Conduct Problems**	0.65	0.88	3.93	3.1	0.21	0.55	26.347	0.001	0.37
**Inattention-Hyperact.**	3.6	1.43	6.9	0.47	1.15	1.5	137.957	0.001	0.75
**Peer Problems**	0.71	1.3	2.1	2.2	0.61	1.29	4.476	0.01	0.09
**Prosocial Behaviors**	7.41	2.65	6.96	1.75	8.45	1.9	2.860	0.062	0.06
**Total Difficulties**	7.1	3.4	16.9	5.2	2.73	2.94	13.642	0.001	0.65

## Data Availability

Data are available on reasonable request from the corresponding author.
